# Strengthening Health Care System in India: Is Privatization the Only Answer?

**DOI:** 10.4103/0970-0218.40869

**Published:** 2008-04

**Authors:** Arun K Aggarwal

**Affiliations:** PGIMER School of Public Health, Chandigarh - 160 012, India. E-mail: aggak63@rediffmail.com

India has achieved substantial improvement in its health indicators. Life expectancy has increased, infant and maternal mortality has declined, and the coverage of most of the National Health Programmes is better. However, this progress is uneven; there are large State-wide variations, and performance in some States is abysmally low.[1] Lack of accountability is plaguing the Indian health system.

The productivity of public health sector has been rather low, and it is often considered one of the ‘sick unit.’ A popular ‘treatment’ to this ‘sickness’ is public-private partnership (PPP), which has become a buzz word today. Although PPP does not imply privatization alone,[2] it has many other options available; but it may lead to privatization in its current format. Providing land and infrastructure to private players and letting them operate the health facilities in their own way cannot be labeled PPP. Monitoring the regulation capacity of public health system is very much inadequate currently, without which PPP is not possible. At this stage, privatization means that 20% of the people who are very poor and depend on government system will be left with no option. In India, already 80% of the curative care is being sought by people from the private sector.[3] Privatization will increase the gap between rich and poor, amounting to encouraging ‘survival of the richest,’ which cannot be the goal of any civilized society. The argument that the poor already incur out-of-the-pocket expense for getting services cannot be applied to favor privatization. Therefore, instead of privatization of health services, one should think of ways and means of using the taxpayers' money, which runs into crores of rupees, to bring the health benefit to the poorest people. Multidisciplinary approach with public health experts taking the lead role can help.

One of the most important functions at State level is resource mapping, planning, and monitoring. Lack of managerial expertise at this level has a cascading effect down the line. State-level health managers require political, administrative, and technical support to initiate effective actions. States should create a health advisory committee with experts from the faculty of public health, business management/health administration institutions, not-for-profit health NGOs, for-profit health organizations, and state health departments. Public health institutions, such us Departments of Preventive and Social Medicine/Community Medicine, can participate in resource mapping, planning, and activity monitoring It will comprise of monthly report reviews from state health departments and client feedback by partner NGOs from consumer groups. Identified problems/issues can then be placed to the committee for finding solutions after taking technical inputs from business management and social science experts. State health officials can facilitate the process. This committee can review district data every three months in a cyclic manner.

Block-wise analysis up to sub-centre level should be done for each district. It is expected that this joint review process will lead to shared understanding of strengths and weaknesses of the health system, activate joint action plans, minimize duplication of efforts, and optimize scarce resources. Computerization of the health service input and output data according to the institutions shall be a primary requirement to identify better performing institutions/individuals based upon agreed minimum indicators for strengthening accountability in the system.

Human resources is another key component of any system. Therefore, incentivization of the human resource should be taken up as a priority issue, i.e. increments/promotions/study leaves, and resource allocation should be linked to performance. Autonomous hospitals need to be created where transfer is not possible, and the staff has a stake in the development of the institution and competes for resources based on the services rendered to the poor people. Recruitment and placement of staff at these institutions should be done at local level on tenure contracts so as to minimize vacancies.

A separate public health cadre at district level should be created with suitable avenues for upgradation of educational qualification to postgraduate level, i.e., Master of Public Health and MD in Community Medicine. They should be entrusted with the management of National Health Programmes for TB, HIV, RCH, etc., and health institutions. Financial management should also receive priority, and financial specialists should be made responsible for budgets, accounting, and auditing of performance. Simple, easy-to-understand MIS linked to management decisions is crucial for public health programs.

Partnership with not-for-profit NGOs has also gradually evolved from that of advocacy to actual partnership in service delivery and quality monitoring since Seventh Five-Year Plan. The government is even willing to hand over the government infrastructure to NGOs or other forms of people's groups for providing health care to the masses within the assigned budgetary provision. This option can be tried in select blocks by contracting the services to not-for-profit NGOs and Departments of Preventive and Social Medicine/Community Medicine by providing the budgets, which will be spent by the state government while, at the same time, giving freedom to hire staff on contract/deputation. State agencies should involve independent public health institutions for monitoring and evaluation of these activities.

The scope of networking with public health institutions that are working in the public sector needs to be expanded within the ambit of the PPP model under the National Rural Health Mission. Handing over public health sector to private hands gradually may not be the right solution.
